# Shielding verification and neutron dose evaluation of the Mevion S250 proton therapy unit

**DOI:** 10.1002/acm2.12256

**Published:** 2018-02-22

**Authors:** Michael T. Prusator, Salahuddin Ahmad, Yong Chen

**Affiliations:** ^1^ University of Oklahoma Health Science Center Stephenson Oklahoma Cancer Center Oklahoma City OK USA

**Keywords:** Monte Carlo, neutron, proton, shielding

## Abstract

For passive scattering proton therapy systems, neutron contamination is the main concern both from an occupational and patient safety perspective. The Mevion S250 compact proton therapy system is the first of its kind, offering an in‐room cyclotron design which prompts more concern for shielding assessment. The purpose of this study was to accomplish an in‐depth evaluation of both the shielding design and in‐room neutron production at our facility using both Monte Carlo simulation and measurement. We found that the shielding in place at our facility is adequate, with simulated annual neutron ambient dose equivalents at 30 cm outside wall/door perimeter ranging from background to 0.07 mSv and measured dose equivalents ranging from background to 0.06 mSv. The in‐room measurements reveal that the H*/D decreases when the distance from isocenter and field size increases. Furthermore, the H*/D generally increases when the angle around isocenter increases. Our results from in‐room measurements show consistent trends with our Monte Carlo model of the Mevion system.

## 1 INTRODUCTION

Proton beam radiation therapy, first introduced in 1946 by Robert Wilson,^1^ is increasing in popularity in both the United States and the rest of the world. Possible benefits of proton therapy lie within the “Bragg Peak,” where at a finite depth, the ionization rises sharply to a maximum and then falls quickly to near zero. This characteristic can offer high‐conformity treatments and sparing of tissues distal to the target in comparison with conventional photon therapy. Advancements in present technology have led proton therapy to be considered as a viable and possibly improved means of providing radiotherapy to a variety of treatment sites.

Hazardous neutrons resulting from intranuclear cascade interactions from incoming protons are highly penetrating in nature and can have high RBE values.^2^, ^3^ Thus, neutron production is the main concern regarding shielding applications and in‐room contamination dose to a patient during the treatment. Typical neutron shielding for proton therapy systems is usually done using one of two accepted methods. One of these two procedures is an analytical method assuming point beam losses, based on the Moyer model,^4^ shown in eq. (1); the other requires full Monte Carlo simulation.

(1) $$H=\frac{H_{0}}{r^{2}}{\left[\frac{E_{p}}{E_{0}}\right]}^{\alpha }exp\left[-\frac{d}{\lambda }\right]$$

where H is the maximum dose equivalent rate at a given radial distance r from the source of neutron production, d is the shield thickness, λ is the attenuation length of the shielding material $E_{0}$ = 1 GeV, $H_{0}$ = 2.6 × 10^−14^ Sv m^2^, deemed the source term, and α is a fitting parameter, fixed at 0.8.

The Moyer model was first introduced as a method for shielding design of high‐energy particle accelerator facilities.^4^ The drawback from the Moyer model, as it is presented in eq. (1), is the lack of flexibility for use in other types of shielding applications. Two major assumptions in this model are that λ and $H_{0}$ are fixed values, which is valid at high energies.^5^ Much work has been done since the introduction of this model to increase its validity for use in shielding design for proton treatment facilities.^6^, ^7^, ^8^, ^9^ The use of analytical models for proton‐shielding applications is well suited for bulk design of a facility, where high efficiency of results can be obtained.^5^

The second shielding calculation method is a full‐scale Monte Carlo simulation of the treatment facility geometry with detectors at points of interest to score dose values. In comparison studies of the Monte Carlo method and the analytical method, the Monte Carlo method was more accurate in situations where the neutron fields were incident on barriers at the angles of high obliquity when compared to measured data.^10^ Shielding studies utilizing complete three‐dimensional Monte Carlo simulations for proton therapy facilities show that Monte Carlo simulations can reasonably predict dose rates at points of interest when compared to measurements.^2^ Newhauser et al. show that simulations agree with experimentally measured neutron dose values to within 10%, with the Monte Carlo values generally overestimating the measured values.^2^ The drawback of this technique was the limitation of computing power required to obtain statistically relevant data at each point of interest in a reasonable amount of time.^10^

In 2012, a new Monte Carlo Code specific for use in proton therapy was introduced. TOPAS, or TOolkit for Particle Simulation, is a Monte Carlo‐based platform that was designed to handle the complexities in geometry introduced by proton therapy systems.^11^ The concept of TOPAS is to preserve the GEANT 4 code as the underlying platform and includes additional codes adapted specifically for proton therapy. TOPAS in proton therapy has the complex geometries prebuilt into the coding toolkit. These include range modulator wheels based on commercially available wheel designs and complex patient apertures and compensators.^11^ TOPAS also has a “time feature” component for the simulation of moving geometries. This component consists of parameters describing the change in a “time feature value,” such as the rotation of the range modulator wheel.

The commercially available Mevion S250 compact proton therapy unit has recently been installed at our institution. Thorough shielding assessments were firstly performed in this study. The Mevion S250 is a compact proton therapy unit in which all components of the treatment system are located in the treatment room. The cyclotron is attached to a gantry that rotates around the patient during the treatment to provide beam at various angles.^12^ The integrity of the shielding put in place must thus be carefully assessed. The unit offers 24 different beam configurations categorized into large, deep, and small groups, each with their own specific beam line components. The large group allows for field sizes up to 25 × 25 cm^2^ and a maximum depth and modulation width of 25 and 20 cm. Both deep and small groups offer a maximum field size of 14 × 14 cm^2^. The deep group has a maximum depth and modulation width of 32 and 10 cm, while the maximum depth and modulation width of the small group are 20 and 20 cm. Both analytical and Monte Carlo methods will be utilized in verifying the adequacy of the radiation shielding design for the Mevion S250 Proton vault at our facility and will be compared with measured data.

The in‐room cyclotron design of the Mevion system provides a unique neutron signature around the patient that has not yet been fully described in a treatment setting. Neutron production from passive scattered proton systems has been extensively investigated, but the conclusions drawn are from units other than Mevion S250.^13^, ^14^, ^15^, ^16^ Chen et al. recently performed a comprehensive neutron assessment of the Mevion S250 using Monte Carlo methods. However, the study was conducted in a factory testing vault with dimensions about two times smaller than the actual treatment vault found at centers that currently use the Mevion system. According to the conclusions drawn from this study, the smaller vault likely confounded their results by an overestimation of the actual in‐room neutron dose.^17^ The second objective of the present study has been to perform a comprehensive neutron dose measurement in a realistic clinical treatment vault.

## 2 METHODS

### 2.A Shielding assessment

A defining characteristic of the MEVION S250 proton system is that the compact superconducting cyclotron, mounted on a rotating gantry, is present in the treatment room with patients. As this single room design has a smaller footprint, challenges arise for radiation shielding. In this study, neutron production from the cyclotron was taken into account as an additive source in the treatment vault.

A simple approach was implemented to model the cyclotron in TOPAS with the assumption that neutrons within the cyclotron primarily were produced from collisions of protons with the magnet and with the extracting foil when exiting. A cylindrical iron target (radius = 5.8 cm, length = 8 cm) was simulated and bombarded with primary proton beams to model a source of neutron production as described in the work of Chen and Ahmad.^18^

The second neutron source considered in this study was the beam delivery nozzle. Passive scattering treatment nozzle structures were built in TOPAS, including first scattering foil, range modulator wheel, the post absorber, and the secondary scattering foil.^19^ The last neutron source modeled in this study was the patient itself. A cubic water phantom (30 × 30 × 40 cm^3^) was placed at machine isocenter to mimic patient body.

The physics list in TOPAS was set to the GEANT 4_Modular physics list that has shown to work well when simulating proton therapy.^11^ “EM‐Standard” was used to model the gamma, electron, and proton electromagnetic processes. Hadron inelastic collisions of protons and neutrons were simulated using the “Binary Cascade” model, and hadronic ion (Z > 1) inelastic collisions were modeled using “Ion‐Binary Cascade.” Elastic collisions of protons, neutrons, and ions were simulated in the “Elastic” model. To model radioactive decay processes, “G4 Decay” was used. The cutoff step range for electron, proton, and gamma was set to 0.05 mm.

Neutron fluences and energy spectra for each of the three neutron sources were scored using a spherical geometry with 10° theta intervals from 0 to 180°, and 1 MeV energy increments up to 250 MeV as was done by Chen and Ahmad.^18^ The fluence at each angle was normalized per incident proton by the total number of incident protons through the target. The ambient dose equivalent, $H^{*}$ per incident proton, for a given energy, E, was calculated by multiplying the particle fluence, Φ(E), with the fluence to dose conversation factors, f(E), from ICRP report number 74.^20^

**Figure 1:**
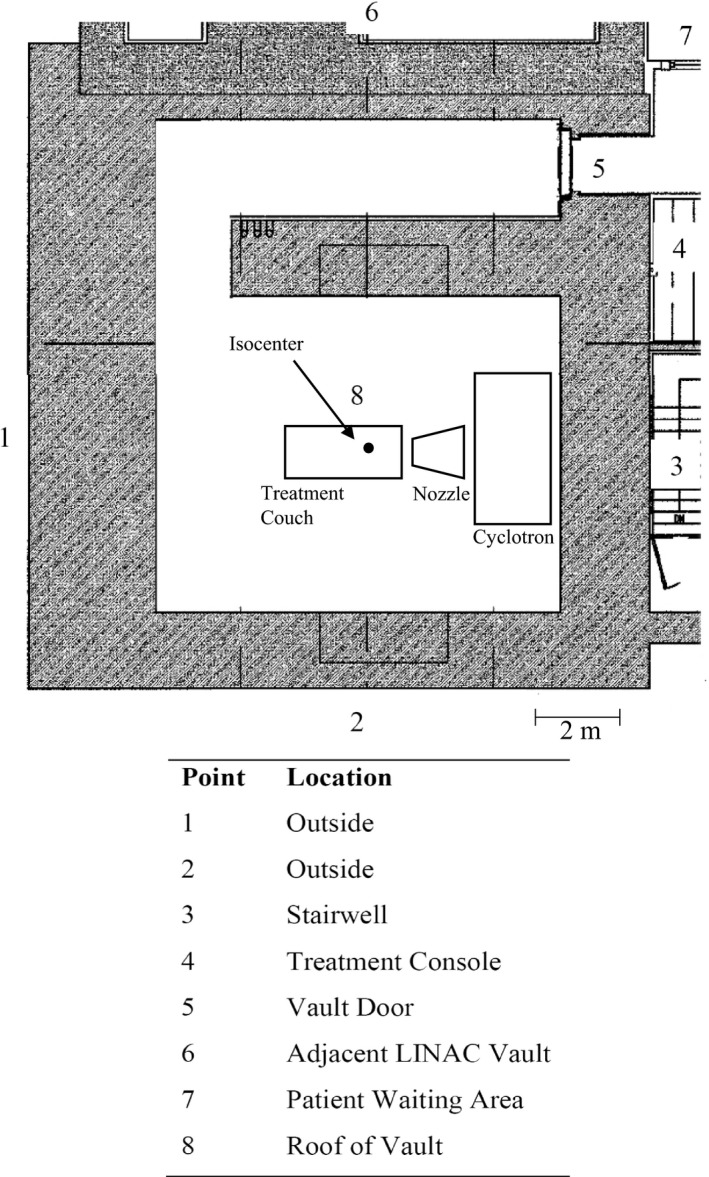
The locations and summary of the eight critical point locations where neutron doses were calculated and measured.

Occupancy, the respective distances, and the angles with respect to the center of the neutron targets for points of interest beyond the barriers of the proton vault were determined utilizing the architectural blueprints provided by the vault construction contractors for our facility. As the cyclotron is located in the room and rotates as the gantry turns, the gantry angle tends to play a role in the measurement of neutron ambient dose equivalent. The angles chosen for calculations were determined that maximum source terms were obtained, as to generate a conservative estimate. In most cases, this resulted in a gantry angle of 0°. A summary of the eight most critical points is shown in Fig. 1.

The workload was calculated based on 20 patients being treated with 2 Gy fractions per day. Under the assumption of adding equivalent treatment‐specific QAs, 40 beams delivering 2 Gy/per beam to isocenter were utilized per day in the calculation. Over a 5 day workweek, 400 Gy of beam was assumed to be delivered by the proton system to isocenter, resulting in a workload of 400 Gy/wk.

As each neutron source contributed dose to the point of interest, eq. (1) was calculated to achieve the ambient dose equivalent per week at each point of interest.

(2) $$H=\left(\mathrm{WT}\right)\sum_{x_{1}}^{x_{3}}N_{Gy,x}^{p}H^{*}{\left(\theta \right)}_{x}\frac{1}{r_{x}^{2}}e^{-d/d\lambda {\left(\theta \right)}_{x}}$$

In the above equation, W was the workload, T was the occupancy factor, $H{\left(\theta \right)}_{x}$ was the source term for one of the three specific sources “x” in pSv/proton, r_x_ was the distance from the specific source to the point of interest, d was the thickness of the shielding material, $\lambda {\left(\theta \right)}_{x}$ was the attenuation length of the shielding material specific to each source, taken from the work of Agosteo et al.,^12^ and $N_{Gy,x}^{p}$ was the number of proton transported through each source to deliver one Gy to phantom. $N_{Gy,x}^{p}$ was adjusted for beam intensity lost in each neutron source and is the factor to convert $H^{*}{\left(\theta \right)}_{x}$ in terms of Gy. The beam losses were assumed to be 10% at the cyclotron (iron target), 10% at the treatment nozzle, and 100% in the water phantom, as was the case in similar previous studies.^21^

For verification of our shielding method, neutron ambient dose equivalent (*H* ^*^) measurements were done at the same eight locations using the SWENDI‐2 (Thermo Scientific, MA, USA) neutron detector. A 250 MeV beam with a range of 15 cm and modulation width of 10 cm delivered proton dose (*D*) to a solid water block phantom measuring 30 × 30 × 40 cm^3^. The MU rate used for measurement was 127 MU/min, and the machine was allowed to deliver dose until a detectable integral neutron ambient dose equivalent was recorded. In many cases, this required 1000 MU to be delivered. The therapeutic dose delivered to the phantom was calculated using the absolute machine output for the given configuration and the actual monitor units delivered.

### 2.B In‐room neutron measurements

**Figure 2:**
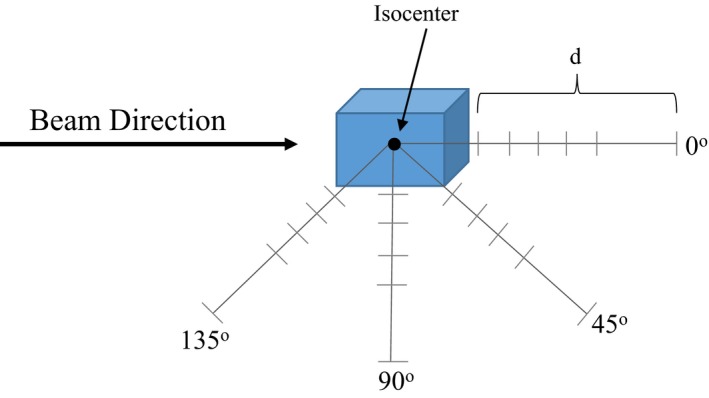
Representation of the points of measurement around the phantom. *d* represents the distance of the detector from the proximal phantom surface. Each tick mark represents a point where a measurement was taken.

Using the SWENDI‐2 neutron detector, in‐room measurement of neutron ambient dose equivalent per therapeutic dose delivered (*H* ^*^ */D*) was made around a 30 × 30 × 40 cm^3^ solid water phantom using a 250 MeV beam at distances of 20, 40, 60, 80, 100, and 150 cm from isocenter. The SWENDI‐2 detector was chosen for the in‐room measurements due to the wide energy response (thermal to 5 GeV) and short dead time.^22^ At these same distances, measurements were done at angles of 0, 45, 90, and 135° around isocenter for a large, deep, and small beam configurations. Figure 2 demonstrates our setup and Table 1 gives a summary of the beam configurations utilized. Under the aforementioned conditions, we employed a full‐scale simulation model, with the concrete vault included, to predict neutron dose values. The MC model from the work of Prusator et al.^19^ were used to model all field‐shaping devices and the nozzle of the Mevion system. Due to the complexity, the cyclotron was not modeled in the simulation but included in the neutron dose analysis via addition of the source term from the iron target in the previous section.

**Table 1:** Beam characteristics of the three configurations chosen for in‐room measurements

Configuration	Range (cm)	Modulation width (cm)
Large	25	20
Deep	30	10
Small	15	10

Using the same beam configurations, neutron measurements were done with different field sizes. Field sizes with diameters of 0, 5, 10, 12, and 15 cm were used for the small and deep configurations and 0, 8, 16, 20, and 26.5 cm for the large configuration. These measurements were also done at a distance of 100 cm from isocenter.

To attain the *H/D* value at each point, dose was delivered to isocenter until an instant neutron dose equivalent rate was observed to be stable via a camera from outside the proton vault. Several measurements were taken at low‐dose (about 20 MU/min) and high‐dose rates (150 MU/min) to assure that the results were not affected by pulse pile up. Assuming the proton dose rate delivered can be calculated using the dose/MU coefficient, the *H/D* is calculated using eq. (2).

(3) $$H/D=\frac{H_{r}}{MU_{r}\;*\;c}$$

where $H_{r}$ is the measured neutron equivalent dose rate in mSv/hr, and $MU_{r}$ is the proton output rate, in MU/hr, and c is the dose/MU calibration coefficient.

## 3 RESULTS AND DISCUSSION

### 3.A Shielding assessment

The ambient dose equivalents for all three targets were normalized at 1 m away from the center of the individual target at 10° increments with respect to the incoming proton beam, ranging from 0 to 180°, and are shown in Table 2. The greatest source of neutron production for our system was the cyclotron, followed by the treatment nozzle and water phantom. These normalized ambient dose equivalent values acquired through simulation were used as the source terms for the subsequent shielding calculations. Our results show that for each individual target, the largest ambient equivalent dose was in the forward direction. This is due to the forward peaked nature of the intranuclear cascade interactions that occurred between the incoming proton and the nucleus of the target atoms.

**Table 2:** Source terms normalized to 1 m from source, at angular increments with respect to the incoming proton beam

H* Sv*m^2^/p			
Angle	Nozzle	Water phantom	Fe target
0–10	2.17E‐15	1.64E‐15	4.96E‐15
10–20	1.24E‐15	1.25E‐15	4.67E‐15
20–30	1.40E‐15	1.35E‐15	4.17E‐15
30–40	1.45E‐15	1.36E‐15	3.62E‐15
40–50	1.45E‐15	1.26E‐15	3.15E‐15
50–60	1.51E‐15	1.14E‐15	2.82E‐15
60–70	1.58E‐15	1.02E‐15	2.61E‐15
70–80	1.50E‐15	9.06E‐16	2.42E‐15
80–90	1.32E‐15	7.92E‐16	2.25E‐15
90–100	1.01E‐15	6.86E‐16	2.17E‐15
100–110	1.18E‐15	5.97E‐16	2.19E‐15
110–120	1.25E‐15	5.27E‐16	2.22E‐15
120–130	1.26E‐15	4.59E‐16	2.26E‐15
130–140	1.25E‐15	4.11E‐16	2.32E‐15
140–150	1.25E‐15	3.66E‐16	2.36E‐15
150–160	1.25E‐15	3.38E‐16	2.42E‐15
160–170	1.27E‐15	3.31E‐16	2.43E‐15
170–180	1.24E‐15	4.35E‐16	2.44E‐15

Table 3 shows the annual doses calculated and those projected from measurement using the SWENDI at each of the eight critical points of interest around the proton treatment vault. The shielding design goals recommended by the NCRP at each point are listed for comparison.^23^ The highest dose calculated was at the treatment console at point 4, with the lowest dose at the waiting area. With regard to the measured neutron doses, the highest and lowest points were in agreement with that from the calculated data. There is reasonable agreement between the calculated and measured values, with the largest discrepancy outside the vault at point 2. In most cases, the calculated values overestimated that what was measured. The differences found in this study may suggest that the analytical method could overestimate the neutron dose, especially when the simplified source terms and attenuation lengths were used. Furthermore, the lower values in measurements may be due to the restrictions on the actual beam‐on time as well as the allowable dose rate. The authors would also like to address that the overall low neutron doses from both simulation and measurement could be contributed by the extra thick barrier wall used in our vault construction. During the shielding survey measurements, the SWENDI detector was set to the integral mode (instead of the rate mode) to accumulate the neutron dose due to the ultra‐low signal strength. The machine parameters were set to the maximum allowable dose rate with 15 min of constant beam on time. Because of the time limit and low dose rate achievable with the unit, it proved difficult to accumulate an accurate appreciable dose. It is also important to note that the workload and occupancy factors were a direct multiplier of the dose beyond the barrier and must be accurately approximated for validation of the results.

**Table 3:** Annual neutron equivalent doses calculated and measured for each of the eight critical points

Pt.	Gantry angle (Degrees)	Occupancy factor (T)	Calculated (mSv)	Measured (mSv)	Design goal (mSv)
1	90	0.0625	0.003	0.001	1
2	0	0.0625	0.05	0.006	1
3	0	0.25	0.06	0.06	1
4	0	1	0.07	0.06	5
5	0	0.25	0.07	0.02	5
6	0	1	0.003	0.001	5
7	0	0.25	Minimal	Minimal	1
8	180	0.025	0.002	0.001	1

### 3.B In‐room measurements

**Figure 3:**
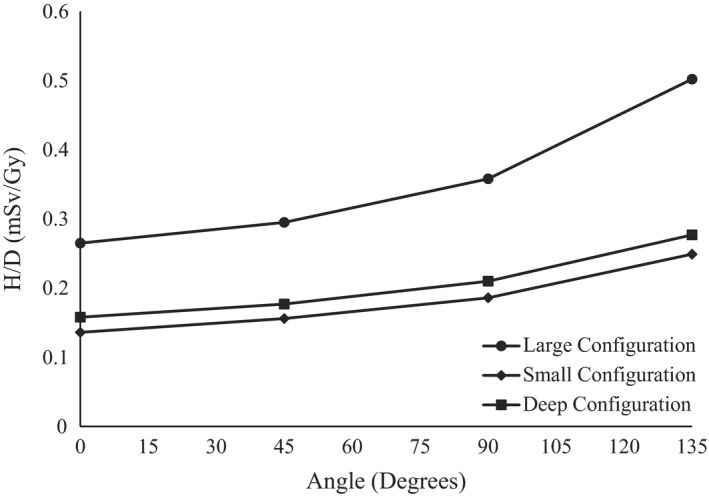
Comparison of H/D vs distance at an angle of 90° between the large, small, and deep configurations.

For the deep and large configurations at all angles around the isocenter, our measurements showed that as the distance from the isocenter increased, the neutron dose equivalent decreased. However, for the small configuration, and at an angle of 135°, the neutron dose equivalent increased from a H/D of 0.22 to 0.26 mSv/Gy. This can possibly be explained with the following: at 135° the distance increased from isocenter. However, the detector was moved closer to the cyclotron and nozzle, which were the main producers of neutrons for shallow ranges due to the presence of energy‐degrading materials upstream of isocenter. Figure 3 shows the measurement results.

**Figure 4:**
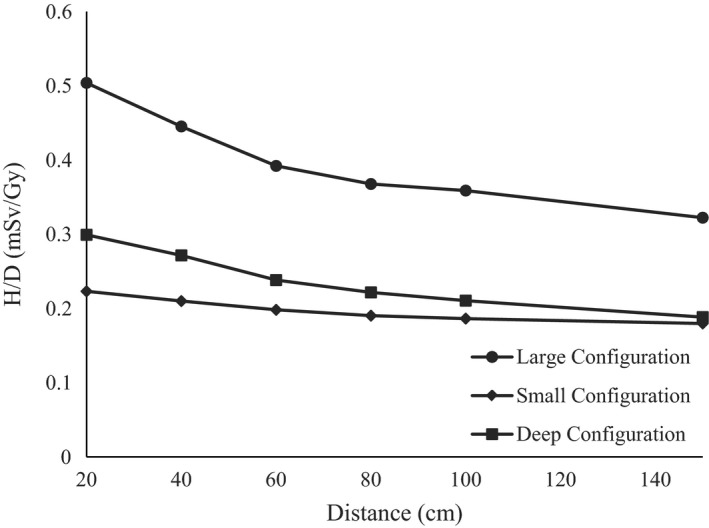
Comparison of H/D vs angle at a distance of 100 cm between the large, small, and deep configurations.

With regard to H/D as a function of angle around the isocenter, the measurements showed for all configurations that the neutron dose equivalent increased as the angle increased. This was likely because increasing the angle brought the detector closer to the neutron‐producing components of the treatment unit, especially the cyclotron. The maximum H/D was found to be, for the large option, at an angle of 135°, with a value of 0.5 mSv/Gy and the minimum value was at 0° for the small option, measuring 0.2 mSv/Gy. A summary of the measurements can be seen in Fig. 4.

**Figure 5:**
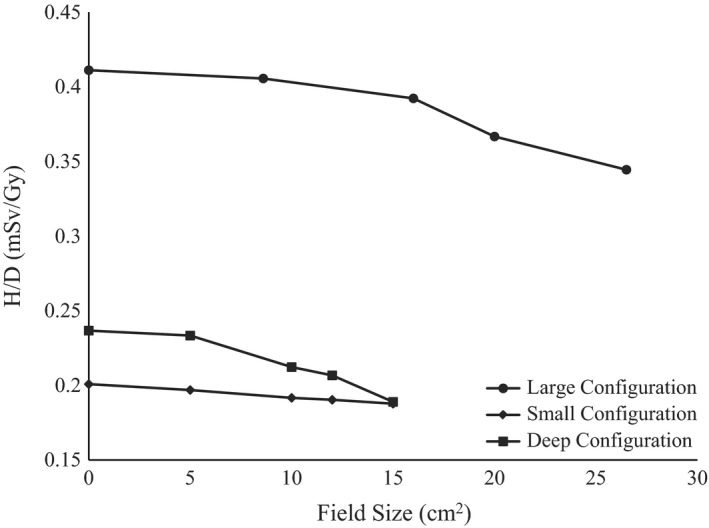
Measured H/D as a function of field size for the three configurations at a distance from isocenter of 100 cm.

For each configuration, as expected, the H/D decreased as the field size increased, as shown in Fig. 5. This was due to the fact that smaller field sizes required larger aperture collimation, resulting in more beam striking brass aperture followed by more neutron production. For this reason, the large option with the smallest field size resulted in the highest H/D of 0.41 mSv/Gy, and the smallest H/D of 0.19 mSv/Gy was created by the small option with the largest field size.

When compared to our simulation results, the doses measured inside the vault followed the same trends but were about an order of magnitude lower. At 1 m and at an angle of 90°, the simulated values for the large, small, and deep configurations were 2.2, 2.0, and 2.2 mSv/Gy, respectively. We believe that this maybe because of the simplistic approach of modeling the cyclotron. The cyclotron was represented as a single iron cylindrical target, and it was assumed that all neutron production came from point losses within the target. In reality, the transport of protons within the cyclotron would result in neutron production throughout the accelerator, thus confounding the assumption of the cyclotron as a point source. Furthermore, self‐shielding is not taken into account when a simple iron target is used to model the accelerator. This, in turn, will cause an over estimation of the neutron contribution from the cyclotron.

## 4 CONCLUSION

All points in controlled and uncontrolled areas predicted by both our calculation model and measurements were beneath the NCRP recommended shielding design goals. The shielding barrier of the proton vault at our facility was found to be sufficient. We believe that because our simulation model overestimated the annual neutron ambient dose calculations in each of the eight points compared to measurements, our model can thus be used as a conservative approach of calculation to verify shielding of a proton facility. Our in‐room measurements generally followed the same trends found through Monte Carlo simulations. In summary, we believe that this data may be used to conduct further study in calculating secondary neutron dose to patients during proton treatments using the Mevion S250 system.

## CONFLICTS OF INTEREST

The authors have no relevant conflicts of interest to disclose.
